# Editorial: Natural diversity in the new millennium

**DOI:** 10.3389/fpls.2015.00897

**Published:** 2015-10-29

**Authors:** Joanna M. Cross, Chiarina Darrah, Nnadozie Oraguzie, Nourollah Ahmadi, Aleksandra Skirycz

**Affiliations:** ^1^Horticulture Department, Inonu UniversityMalatya, Turkey; ^2^Eunomia Research and ConsultingBristol, UK; ^3^Irrigated Agriculture Research and Extension Center (IAREC), Washington State UniversityProsser, WA, USA; ^4^Centre de coopération internationale en recherche agronomique pour le développementMontpellier, France; ^5^Instituto Tecnológico Vale Desenvolvimento Sustentável/Vale Institute of Technology Sustainable DevelopmentBelem, Brazil

**Keywords:** natural diversity, crop improvement, next generation sequencing, biodiversity, plant biotechnology

Natural diversity is a recurrent theme in everyday life and in research. Thus, the objective of this topic was to highlight progress or novelties that occurred in this new millennium. The topic includes many different fields as can be seen by the variety of the articles. Indeed, subjects covered include variations in strawberry aroma, diversity of protein families, and peculiar ecosystems. However, several themes connect this apparently unrelated set of articles.

Unfortunately, one cannot ignore the issue of biodiversity and loss thereof. The United Nations Climate Change Conference was held in Copenhagen in 2009 to discuss ways of mediating environmental changes. No simple agreement was signed by all participating countries. That, combined with press articles has left a negative feeling. Thus, the general opinion is that the world is divided into two, the Western group environmentally friendly, and the rest of the world environmentally unfriendly. The articles reveal a more complex picture as many countries are trying to combine sustainable industries with economic realities. For instance, the Brazilian government has issued laws to safeguard the peculiar Canga ecosystems while allowing mining (Skirycz et al., 2014). Efforts are underway to produce improved oil palms to spare land (Barcelos et al., 2015). Moreover, many ecological studies can be found in the literature which highlights the international concern for loss of biodiversity. Unfortunately, biodiversity loss also applies to crops. Many landraces are abandoned as farmers adopt a few high yielding varieties. This results in a significant loss of genetic variability. Thus, more diversity was found in a disease resistance gene in rice landraces than in common varieties (Thakur et al., 2015). Once again, the articles illustrate an emphasis on collecting cultivars even for orphan crops. For instance, the Ethiopian Institute of Biodiversity houses 5000 accessions of tef, a major staple crop for the country (Assefa et al., 2015). The number represents a four-fold increase over the last 20 years. Likewise, significant resources exist for millet (Goron and Raizada, 2015).

Assessment of biodiversity requires a combination of phenotypic and molecular techniques. Technological improvements have been significant in all areas. Hence, metabolomics can now detect many complex molecules such as aroma compounds. As a result, factors determining the taste of strawberry (Negri et al., 2015) and other fruits can be elucidated. However, the most spectacular change comes from the decrease in cost and enhanced speed of Next Generation Sequencing. Fifteen years ago, international consortiums sequenced a few model species and major crops. Now many universities are acquiring their own sequencing equipment. Most plants covered in the articles harbor some sequencing resources either as transcriptomics or as an annotated genome. This opens enormous possibilities for identifying new genetic variations, assessing the variability of isozymes, and associating a given phenotype with regions in the genome. Thus, sequencing reveals the enormous evolutionary potential of viruses (Huang et al., 2015). In addition, extensive diversity was found in the MAP, MAPP, MAPPP kinase families in grapevine (Çakir and Kılıçkaya, 2015).

Finally, these articles illustrate the spectacular biological diversity of plants. They seem to survive in any environment, by any means. Hence, a seemingly fixed structure such as the cell wall shows remarkable flexibility in response to environmental, physiological, and genetic cues (Parrotta et al., 2015). Moreover, the richness of plant metabolism is further enhanced by symbiotic relations with microorganisms (Mousa and Raizada, 2015). However, while well described, this diversity harbors many mysteries as to biological functions, evolutionary adaptations, and physiological mechanisms. With the enhanced technologies, let's hope we can understand and safeguard our beautiful world.

## Author contributions

JC, CD, and AS contacted participants; JC and AS edited manuscripts; NA and NO partly edited articles.

### Conflict of interest statement

The authors declare that the research was conducted in the absence of any commercial or financial relationships that could be construed as a potential conflict of interest.
